# Gut Microbiota and Cardiovascular Diseases: Unraveling the Role of Dysbiosis and Microbial Metabolites

**DOI:** 10.3390/ijms26094264

**Published:** 2025-04-30

**Authors:** Barathan Muttiah, Alfizah Hanafiah

**Affiliations:** 1 Department of Medical Microbiology and Immunology, Faculty of Medicine, Universiti Kebangsaan Malaysia, Kuala Lumpur 56000, Malaysia; 2 GUT Research Group, Universiti Kebangsaan Malaysia, Kuala Lumpur 56000, Malaysia

**Keywords:** gut microbiota, cardiovascular disease, dysbiosis, trimethylamine-N-oxide, natural products

## Abstract

Cardiovascular diseases (CVDs), including heart failure (HF), hypertension, myocardial infarction (MI), and atherosclerosis, are increasingly linked to gut microbiota dysbiosis and its metabolic byproducts. HF, affecting over 64 million individuals globally, is associated with systemic inflammation and gut barrier dysfunction, exacerbating disease progression. Similarly, hypertension and MI correlate with reduced microbial diversity and an abundance of pro-inflammatory bacteria, contributing to vascular inflammation and increased cardiovascular risk. Atherosclerosis is also influenced by gut dysbiosis, with key microbial metabolites such as trimethylamine-N-oxide (TMAO) and short-chain fatty acids (SCFAs) playing crucial roles in disease pathogenesis. Emerging evidence highlights the therapeutic potential of natural compounds, including flavonoids, omega-3 fatty acids, resveratrol, curcumin, and marine-derived bioactives, which modulate the gut microbiota and confer cardioprotective effects. These insights underscore the gut microbiota as a critical regulator of cardiovascular health, suggesting that targeting dysbiosis may offer novel preventive and therapeutic strategies. Further research is needed to elucidate underlying mechanisms and optimize microbiome-based interventions for improved cardiovascular outcomes.

## 1. Introduction

Noncommunicable diseases like cardiovascular diseases (CVDs), cancers, respiratory diseases, and diabetes cause over 63% of total mortality [1], and CVDs accounted for ~19 million deaths in 2021 [2] alone, followed by cancers (10 million) [3], respiratory diseases (4 million) [4], and diabetes (>2 million) [5]. Tobacco smoking, unhealthy diet, physical inactivity, alcohol consumption, and air pollution are some common risk factors, which affected disproportionately low- and middle-income countries (LMICs) [6]. Chronic diseases will be responsible for 75% of all deaths worldwide by 2030 [7] because of age, physical inactivity, poor diet, and environmental exposure, further weakening health systems [8]. They are overlooked at the global level due to myths, risk underestimation, and a lack of investment in prevention [9]. LMICs struggle with chronic disease prevention due to limited access to care, shortages of trained professionals, and inadequate infrastructure [10], while high-income countries (HICs) face rising healthcare costs, health disparities, and challenges integrating preventive care [11]. Conventional therapies such as drugs, chemotherapy, and immunotherapy have improved disease control but are limited by resistance, side effects, and incomplete efficacy [12], necessitating alternative approaches. Emerging evidence indicates the etiology of metabolic disease by the microbiome through metabolic, immune, and neuroendocrine modulation [13].

Gut dysbiosis may contribute to metabolic diseases, chronic inflammation, and even tumorigenesis through immune dysregulation [14,15]. Chronic infections including *Helicobacter pylori* lead to gastric cancer [16,17], hepatitis B virus/hepatitis C virus (HBV/HCV) to hepatocellular carcinoma [18,19], and Epstein–Barr virus (EBV) reprograms immune pathways leading to lymphoma [20]. These infections induce inflammation, oxidative stress, immune suppression, and genetic/epigenetic alterations [21], resulting in obesity, diabetes, and metabolic dysfunction [22]. Microbiome targeting can enhance existing treatments and curtail disease progression [23]. Natural products like polyphenols (curcumin, resveratrol, and quercetin), probiotics, and microbial metabolites have therapeutic value via microbiota modulation, anti-inflammation, and drug metabolism enhancement [24,25]. The role of the microbiome in chronic disease pathogenesis has been emphasized in recent research, with dysbiosis linked to obesity, type 2 diabetes, inflammatory bowel disease (IBD), and cancer [26,27]. Understanding microbiome-mediated mechanisms could uncover new interventions, including probiotics, prebiotics, and fecal microbiota transplantation. This review is centered on CVDs and microbiome-focused natural products as adjuvants to traditional treatments. Due to the high global load of CVDs and their metabolic and inflammatory associations, it is important to investigate microbiome-based therapies for new treatment options [28].

## 2. Human Microbial Contributions

The gut microbiota is a diverse microbial community that is essential for human health, and is linked with improved metabolism, immune function, and decreased chronic disease risk. It mostly consists of *Bacillota* and *Bacteroidota* which make up about 90% of the microbes in a healthy adult [29]. The microbiota has over 1000 species with a predicted 150-fold more genes than the human genome [30]. Each individual has a unique microbiome with less than 10% commonality between two individuals [31]. *Bacillota*, including *Lactobacillus*, *Clostridium*, *Ruminococcus*, and *Faecalibacterium*, are accountable for fiber fermentation and SCFA production, stimulating colonocyte energy and immune control [32]. *Bacillota* are also engaged in energy metabolism, and increased levels are linked with obesity. *Bacteroidota*, including *Bacteroides* and *Prevotella*, are involved in carbohydrate metabolism, SCFA production, and gut barrier integrity [33]. A balanced ratio of *Bacillota* to *Bacteroidota* is critical since its disruption has been associated with metabolic disease [34]. *Actinobacteria*, particularly *Bifidobacterium*, are probiotic, exerting immune modulation and pathogen suppression [35]. *Proteobacteria*, including *Escherichia*, *Enterobacter*, and Helicobacter, occur in low numbers by nature, but overgrowth has been associated with inflammation of the intestine and diseases such as IBD and colorectal cancer [36].

Diet affects gut microbiota diversity by providing nutrients for microbial metabolism, regulating bacterial composition [37]. High-fiber diets, common in non-Western populations, encourage beneficial bacteria like *Prevotella* to ferment complex carbohydrates into SCFAs like butyrate, acetate, and propionate [38]. These metabolites strengthen gut barrier function, anti-inflammatory responses, and metabolic homeostasis. Meanwhile, Western diets, which are rich in fat and processed food and poor in fiber, reduce microbial diversity, promote pro-inflammatory taxa (*Bacteroides*), and lower beneficial bacteria (*Akkermansia muciniphila* and *Faecalibacterium prausnitzii*) and may predispose to obesity, type 2 diabetes, and cardiovascular disease [39]. The polyphenols found in fruit and vegetables have prebiotic actions, promoting beneficial bacteria selectively. High-protein diets, particularly diets high in red meat, increase *Odoribacter splanchnicus* and trimethylamine-N-oxide (TMAO), a metabolite linked to cardiovascular risk [40]. Fermented foods like yogurt, kefir, kimchi, and sauerkraut introduce probiotics (*Lactobacillus* and *Bifidobacterium*), which boost microbial diversity and intestinal health [41]. Other than diet, gut microbiota populations are influenced by several lifestyle, genetic, and environmental determinants [42]. Aging comes with declines in protective bacteria like *Bifidobacteria* and rises in pathogenic taxa that cause inflammation and reduced gut resilience [43]. Exercise raises microbial diversity and SCFA output, but lifestyle sedentism promotes gut dysbiosis [44]. Smoking and alcohol abuse negatively influence microbiota by raising pro-inflammatory bacteria and intestinal permeability [45]. Agents such as antibiotics, PPIs, and NSAIDs cause chronic microbial dysbiosis, which enhances susceptibility to infections such as *Clostridioides difficile* [46]. Genetic predisposition regulates microbial colonization, which affects immune function and metabolic competence [47]. Geographical settings, cultural habits, and environmental toxins (e.g., heavy metals, pesticides, and microplastics) also regulate microbiome composition, potentially leading to inflammation and metabolic disease [48]. Early-life exposures, including birth mode, breastfeeding, and early-life antibiotic exposure, have a profound impact on microbiota diversity, influencing immune development and disease susceptibility [49].

## 3. Cardiovascular Diseases (CVDs) and Gut Microbiota

CVDs are a leading cause of morbidity and mortality worldwide, with multifactorial etiologies rooted in genetic, lifestyle, and environmental determinants [50]. There is growing evidence to support the notion that the gut microbiota, the vast array of microorganisms within the gastrointestinal tract, plays a significant role in modulating cardiovascular health [51]. The host metabolism–microbial composition interaction has been highlighted more and more, particularly in relation to dysbiosis and its contribution to CVD pathogenesis [52]. The gut microbiota plays a critical role in the pathophysiology of CVDs such as atherosclerosis, hypertension, myocardial infarction, and heart failure (HF), through intricate mechanisms involving inflammation, metabolism, and microbial dysbiosis [51].

## 4. HFs and Gut Microbial Changes

Heart failure (HF) is a clinical syndrome of impaired cardiac performance, leading to reduced blood flow. There were over 64 million cases worldwide in 2017; HF remains a significant health emergency, and its prevalence is estimated to rise due to increased survival and aging populations [53]. Its economic burden is also high, with U.S. healthcare spending projected to reach $69.8 billion by 2030 [54].

There is growing evidence that gut dysbiosis is involved in HF progression through the disruption of homeostasis, increased intestinal permeability, and the induction of systemic inflammation [51]. HF-related structural and functional gastrointestinal alterations, including systemic redistribution of blood, reduced cardiac output, and neuro-humoral activation, cause the thickening of the intestinal wall, edema, and barrier dysfunction [55]. This allows for microbial translocation and systemic inflammation, and elevated inflammatory markers are predictive of worse HF prognosis [56]. Also, increased concentrations of bacteria and adhesion to intestinal mucosa further increase inflammation, and HF patients have greater rates of infection with *Clostridium difficile*, which add to inflammatory load [57,58]. Gut microbiota metabolites have significant influences on the pathogenesis of HF.

SCFAs like acetate, propionate, and butyrate have protective effects by promoting intestinal barrier function, modulating immune response, and managing glucose and lipid metabolism. SCFAs activate G-protein coupled receptors (GPR41, GPR43, and GPR109A), leading to immune modulation by upregulating anti-inflammatory cytokines (IL-10) and decreasing systemic inflammation, a characteristic of HF [59]. They also inhibit histone deacetylases (HDACs), which affect gene expression to upregulate anti-inflammatory genes, improve gut barrier function, and prevent bacterial endotoxin translocation, all of which are involved in HF [60]. SCFAs thus maintain the integrity of the gut epithelium and reduce microbial translocation, which promotes additional systemic inflammation and endothelial function [57,58,59].

On the other hand, TMAO, a gut-derived metabolite of dietary choline and carnitine, induces NF-κB activation to induce pro-inflammatory cytokine generation; worsens endothelial dysfunction, oxidative stress, and atherosclerosis; and facilitates the progression of HF [61]. TMAO also alters mitochondrial function and reduces myocardial contractility with cardiac dysfunction [62]. TMAO also activates the ROS/TXNIP/NLRP3 inflammasome and TGF-β1/Smad3 signaling pathways to cause fibrosis and maladaptive cardiac remodeling. Increased TMAO levels are associated with higher HF mortality, and pharmacologic inhibition of TMAO pathways has been shown to prevent the progression of HF [63,64].

Bile acids (BAs), branched-chain amino acids (BCAAs), and tryptophan derivatives are also involved in HF through diverse mechanisms. Bile acids activate the FXR pathway, modulating lipid metabolism and immune responses [65], while BCAAs activate the mTOR pathway, causing oxidative stress and inflammation [66]. Tryptophan metabolites, like indole derivatives, modulate the NLRP inflammasome and the PERK pathway, influencing immune responses, inflammation, and cellular stress, all of which dictate the severity of HF [67]. BAs also experience compositional alterations in HF patients, and a higher secondary-to-primary BA ratio is a predictor of poor survival. BA overload induces cardiac hypertrophy and metabolic derangement by inhibiting PGC-1α and repressing fatty acid oxidation [68]. In addition, excessive intracellular BA accumulation also activates AIM2 inflammasomes and type I interferon pathways, inducing chronic myocardial inflammation, mitochondrial dysfunction, and fibrosis [69].

Another key stimulus of HF progression is gut-derived lipopolysaccharide (LPS). LPS translocation into the circulatory system triggers systemic inflammation, oxidative stress, and fibrosis. LPS signals via Toll-like receptor 4 (TLR4) to trigger cytokine release, arrhythmias, and cardiac injury, whereas oxidative imbalance and MMP-mediated remodeling exacerbate contractility [70]. Gut dysbiosis also sustains systemic inflammation by increasing immune cell infiltration, compromising gut barrier integrity, and increasing inflammatory signaling [71]. With the pivotal role of inflammation in HF development, the pharmacological targeting of inflammatory pathways potentially provides new therapeutic options [70]. The gut microbiota collectively modulates key pathways, emphasizing the gut microbiota’s importance in HF pathophysiology. Table 1 explains the various pathway modulations by metabolites in HF.

## 5. Hypertension and Gut Microbial Changes

Hypertension, or high blood pressure, is one of the world’s most significant public health challenges and a leading risk factor for cardiovascular morbidity, stroke, and kidney disease [72]. It accounts for approximately 8.5 million deaths annually, highlighting the need for urgent action in prevention and treatment. Despite being a preventable and manageable condition, hypertension often remains undiagnosed or inadequately controlled, particularly in low- and middle-income countries [73]. Effective screening, early detection, and cost-effective treatment strategies are crucial in reducing its burden [74]. Recent studies have established a strong connection between gut microbiota dysbiosis and hypertension in both animal models and humans. The gut–brain axis directly influences blood pressure regulation, as microbiota-induced gut alterations can enhance sympathetic drive through either direct intestinal wall nerve–brain interactions or neuroinflammation, consequently modulating central blood pressure control pathways [75]. Compositional changes in the gut microbiota have been linked to increased sympathetic outflow, contributing to elevated blood pressure [76]. Hypertensive individuals often exhibit reduced microbial richness, diversity, and evenness, along with an increased *Firmicutes/Bacteroidetes* ratio [76]. A decrease in acetate- and butyrate-producing bacteria has also been implicated in hypertension [77]. For example, human studies indicate that hypertensive patients have elevated levels of *Klebsiella*, *Streptococcus*, and *Parabacteroides*, whereas beneficial bacteria such as *Roseburia* and *Faecalibacterium* are significantly reduced [78]. Beyond microbial composition, gut dysbiosis promotes systemic inflammation by activating T-helper 17 (Th17) cells and stimulating the release of pro-inflammatory cytokines [79]. This immune imbalance contributes to T-cell infiltration in vascular tissues, leading to inflammation, endothelial dysfunction, and ultimately, hypertension [80]. Additionally, gut microbial metabolites influence critical physiological processes, including energy balance, catecholamine metabolism, and ion transport, all of which impact blood pressure regulation [81]. Mechanistically, gut dysbiosis is associated with an increase in LPS-producing bacteria, leading to low-grade endotoxemia and vascular inflammation [82]. These factors contribute to endothelial dysfunction, a key driver of hypertension. SCFAs stimulate these receptors, notably GPR43, to influence vasodilation, inhibit inflammatory cytokine production, and regulate sympathetic nervous system activity [77]. SCFAs also inhibit components of the renin–angiotensin–aldosterone system (RAS), a central controller of blood pressure [83]. Meanwhile, butyrate increases endothelial nitric oxide synthase (eNOS) expression, increasing the availability of nitric oxide (NO), leading to vasodilation [84]. An emerging mechanism involves the role of olfactory receptors (ORs) in the cardiovascular system, which are influenced by gut microbiota-derived metabolites such as SCFAs. These ORs help regulate blood pressure by modulating sympathetic nervous activity and vascular tone. For example, Olfr78 responds to SCFAs by regulating renin secretion, while OR10J5 is expressed in endothelial cells, further emphasizing the gut–heart connection in hypertension [85]. TMAO is synthesized from dietary substrates like choline, carnitine, and betaine through microbial metabolism to trimethylamine (TMA) that undergoes further oxidation in the liver by flavin monooxygenase 3 (FMO3). TMAO elevation is linked to hypertension through the activation of NF-κB signaling that enhances vascular inflammation and endothelial dysfunction [86]. TMAO increases platelet hyperresponsiveness, increasing the risk of thrombotic events [87]. Tryptophan degradation by gut microbiota yields indole derivatives, kynurenine, and serotonin, all of which exert influences on vascular and renal function. Indole derivatives stimulate the aryl hydrocarbon receptor (AhR), influencing immune responses and maintaining vascular integrity [88]. Polyamines such as putrescine and spermidine either activate or inhibit nitric oxide production, controlling vascular tone and blood pressure [89]. These findings underscore the critical role of the gut microbiota in hypertension and suggest that targeting gut dysbiosis may offer novel therapeutic strategies for its management, as shown in Table 2.

## 6. Myocardial Infarction and Gut Dysbiosis

Globally, myocardial infarction (MI) is a significant cause of death, affecting an estimated 3 million people worldwide, with over a million annual deaths in the US alone [90]. The global burden of MI is 3.8% among individuals younger than 60 years and rises to 9.5% among those older than 60 years. In 2019, MI caused an estimated 8.9 million deaths, accounting for 16% of total global mortality [91]. Risk factors include high blood pressure, smoking, diabetes, physical inactivity, obesity, high cholesterol levels, poor diet, and excessive alcohol consumption [92]. While the incidence of MI is declining in developed countries due to improved healthcare and public health interventions, it is increasing in developing regions such as South Asia, parts of Latin America, and Eastern Europe [93].

Recent research highlights the relationship between the gut microbiota and MI, emphasizing how gut dysbiosis influences cardiovascular health [94]. MI patients exhibit significant alterations in gut microbiota composition, including a decreased *Firmicutes-*to*-Bacteroidetes* ratio and an increased abundance of pro-inflammatory bacteria such as *Desulfovibrio* and *Acidaminococcus* [95]. In acute myocardial infarction (AMI) patients, a distinct microbial shift has been observed, with notable reductions in beneficial genera such as *Prevotella* and *Parabacteroides* known for maintaining gut homeostasis and regulating inflammation alongside increases in genera like *Bifidobacterium* and *Dorea* [96]. These microbial changes correlate with metabolic dysregulation, particularly in lipid metabolism. In early AMI patients, gut dysbiosis has been associated with high concentrations of long-chain fatty acids (LCFAs), which promote platelet aggregation, a critical step in thrombus formation and MI initiation [97]. Additionally, an altered gut microbiota composition contributes to systemic inflammation through elevated LPS levels, which enter the circulatory system and activate inflammatory pathways via TLR4, exacerbating cardiovascular disease and MI outcomes [98]. Beyond inflammation, gut dysbiosis affects lipid homeostasis, glucose metabolism, and vascular integrity, further increasing cardiovascular risk [99]. Notably, LCFAs have been proposed as potential early biomarkers for early AMI (eAMI), underscoring the gut microbiome’s diagnostic potential in cardiovascular disease [98].

Following MI, intestinal permeability increases, allowing gut bacteria to translocate into the circulatory system, exacerbating inflammation. Studies indicate that over 12% of blood bacteria in ST elevation MI (STEMI) patients originate from the gut, correlating with elevated LPS levels that drive systemic inflammation and worsen cardiovascular prognosis [100]. The gut-derived metabolite TMAO is produced from the microbial metabolism of dietary choline and L-carnitine and has been implicated in promoting atherosclerosis and increasing MI risk [101]. Another notable microbial metabolite, phenylacetylglutamine (PAGln), derived from phenylalanine metabolism, influences MI severity by activating adrenergic receptors on platelets, thereby enhancing platelet responsiveness and promoting thrombosis. Elevated PAGln levels are associated with increased thrombotic risk, potentially worsening MI outcomes [102].

Conversely, SCFAs, beneficial microbial metabolites that regulate inflammation and blood pressure, are often reduced in MI-related dysbiosis, potentially impairing recovery. Although plasma SCFA levels may not differ significantly in acute MI patients, fecal SCFA profiles often show alterations, reflecting microbial shifts during MI. Additionally, aromatic amino acid metabolites have been linked to oxidative stress and myocardial infarct size, further influencing post-MI outcomes [103].

Gut dysbiosis in MI patients triggers several pathological pathways. Firstly, inflammation and immune activation are enhanced because TMAO and LPS of gut microbiota stimulate NF-κB signaling, leading to the release of pro-inflammatory cytokines (TNF-α, IL-6, and IL-1β), which enhance myocardial injury and inhibit cardiac repair [104]. Secondly, TMAO and LPS promote oxidative stress and endothelial dysfunction by augmenting ROS production, disrupting eNOS activity, and reducing NO bioavailability, worsening ischemic and reperfusion damage [105]. Thirdly, calcium signaling and cardiac contractility are disrupted, whereby TMAO augments the intracellular calcium level, deranging excitation–contraction coupling, disrupting myocardial contractility, and elevating arrhythmogenicity [106]. Also, MI-associated gut hypoperfusion and ischemia result in compromised gut barrier integrity (“leaky gut”) allowing the entry of microbial products like LPS into the circulatory system, which worsens systemic inflammation and myocardial damage [107]. Understanding the intricate relationships between gut microbiota, inflammation, and cardiovascular health remains a critical research area, offering insights into innovative microbiome-based strategies for MI prevention and treatment in Table 3.

## 7. Atherosclerosis and Gut Microbiota Composition

Atherosclerosis, which is an inflammatory disease of large and medium-sized arteries that advances progressively, is a major cause of ischemic heart disease (IHD), ischemic stroke, and peripheral arterial disease (PAD) [108]. Despite spectacular reductions in IHD- and stroke-related mortality in rich countries since the middle of the 20th century, IHD is the single most significant cause of early adult death in the world [109]. Low- and middle-income countries, in contrast, have reported varied trends where a few nations recorded decreased stroke mortality, but others continue to be affected by rising IHD mortality [110].

The pathogenesis of gut dysbiosis and its contribution to atherosclerosis is characterized by distinctive microbial patterns and metabolic derivatives that directly influence cardiovascular health [111]. Notably, studies have identified distinctive microbial alterations that are associated with atherosclerosis. Pathogenic bacteria such as *Megamonas, Veillonella*, and *Streptococcus* are found in higher concentrations in atherosclerosis patients, whereas the beneficial genera *Bifidobacterium* and *Roseburia*, which exert anti-inflammatory and protective actions on the gut, are significantly reduced [112]. In addition, fungal dysbiosis, encompassing high *Candida albicans*, has been linked with dyslipidemia and atherosclerosis development via the modulation of the HIF-2α–ceramide pathway, which indicates the cardiovascular disease function of the gut mycobiome [113]. Patients with atherosclerotic cardiovascular disease (ACVD) exhibit pronounced alterations in gut microbiota profiles. All the studies describe elevated levels of pro-inflammatory and potentially pathogenic bacterial taxa, such as *Enterobacteriaceae* (with *Escherichia coli*), *Streptococcus* spp., *Eggerthella*, and *Ruminococcus gnavus*. These bacteria are associated with elevated inflammatory potential and systemic immune activation [114].

Beyond microbial composition, the metabolic byproducts of the gut microbiota are mediators that have a prime function in atherosclerosis development. High-fat diets can induce gut dysbiosis, compromising intestinal integrity and promoting atherosclerosis through metabolism-independent and metabolite-dependent pathways [115]. Moreover, gut lymphocytes affected by diet-induced dysbiosis proliferate in mesenteric lymph nodes and migrate to atherosclerotic plaques, fueling T-cell accumulation [116].

TMAO is a gut microbiota metabolite produced from dietary precursors like choline, phosphatidylcholine, and L-carnitine. TMAO pathways are key inducers of atherosclerosis, and elevated TMAO impairs the bioavailability of nitric oxide (NO), a molecule central to endothelial function and vascular health. This leads to endothelial dysfunction, a key early event in atherogenesis [117]. TMAO also enhances foam cell formation, which is a key process in the formation of atherosclerotic plaques. Foam cells are macrophages laden with lipids that accumulate in arterial walls, leading to plaque expansion and instability [118]. In addition, TMAO also triggers mitogen-activated protein kinase (MAPK) and nuclear factor kappa B (NF-κB) signaling pathways, both of which are involved in inflammatory responses. The resulting oxidative stress and inflammation destabilize the endothelial lining further and cause plaque rupture [119]. TMAO has also been shown to inhibit the reverse transport of cholesterol, a process by which cholesterol is moved from the peripheral tissues to the liver for excretion. Such inhibition leads to increased deposition of cholesterol in the arterial walls, encouraging atherosclerosis [120].

**Figure 1:**
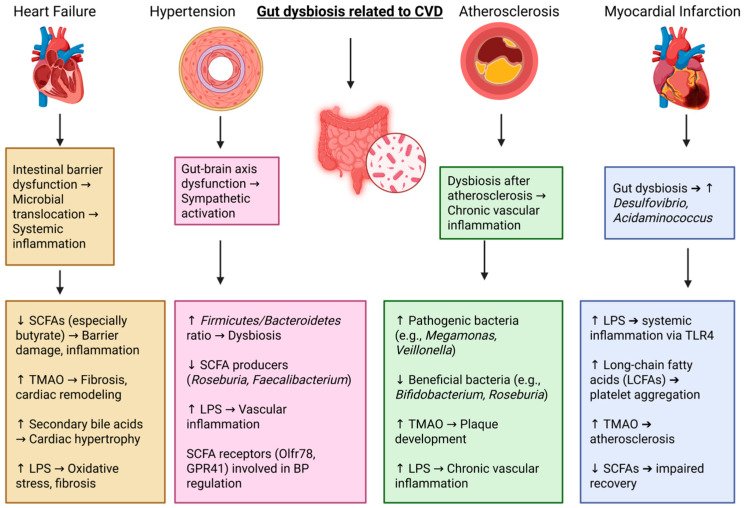
Gut dysbiosis and cardiovascular diseases.

SCFAs, including butyrate, acetate, and propionate, on the other hand, have been reported to have protective roles in the regulation of inflammation, the maintenance of the intestinal barrier, and the regulation of lipid metabolism, specifically by tightening the gut lining, and SCFAs also prevent the translocation of harmful microbial components (LPS) into the circulatory system, thus reducing systemic inflammation [121]. SCFAs also activate peroxisome proliferator-activated receptor gamma (PPARγ), which plays an important part in the control of lipid metabolism and the minimization of inflammation within the vasculature [122]. Nonetheless, dysbiosis tends to be characterized by a considerable decrease in SCFA-producing bacteria, and this causes higher inflammation, weakened immune function, and elevated cardiovascular risk [123]. Table 4 shows the pathways of gut-derived metabolites that orchestrate complex pathological changes during atherosclerosis. In summary, these results highlight the complex interplay between the gut microbiota and atherosclerosis and offer new therapeutic opportunities for interventions into microbial composition and function. Figure 1 describes gut microbiota changes in CVDs (heart failure, hypertension, myocardial infarction, and atherosclerosis); meanwhile, Table 5 explains the impact of the composition of the gut microbiota and its metabolites on various CVDs.

## 8. Natural Products and Their Potential in CVD

CVDs remain the leading cause of morbidity and mortality worldwide, imposing an enormous global health burden. These conditions, such as coronary artery disease, heart failure, and hypertension, are managed with pharmacological agents, surgery, and lifestyle modification [124]. Statins (Atorvastatin, Rosuvastatin, and Simvastatin) lower LDL cholesterol to restrict atherosclerosis [125], while beta-blockers (Metoprolol, Carvedilol, and Bisoprolol) decrease the heart rate and blood pressure, reducing cardiac workload [126]. Angiotensin-converting-enzyme (ACE) inhibitors (Enalapril, Lisinopril, and Ramipril) and ARBs (Losartan, Valsartan, and Candesartan) control blood pressure and avoid cardiac remodeling [127]. Antiplatelets (Aspirin, Clopidogrel, and Ticagrelor) and anticoagulants (Warfarin, Rivaroxaban, and Apixaban) avert clot formation and stroke, respectively [128]. PCSK9 inhibitors (Alirocumab and Evolocumab) further reduce LDL cholesterol by allowing receptor recycling, while SGLT2 inhibitors (Empagliflozin, Dapagliflozin, and Canagliflozin) increase glucose excretion and reduce cardiovascular events in diabetes [129]. Surgical interventions such as Coronary Artery Bypass Grafting (CABG) restore blood flow in ischemic heart disease [130], and Transcatheter Aortic Valve Replacement (TAVR) with artificial valves (e.g., Edwards Sapien) and mechanical or biologic valve replacements manage heart valve dysfunction [131]. In addition to surgical and medical treatment, lifestyle modification, including adherence to heart-healthy diets like the Mediterranean diet and DASH diet, and attending formal cardiac rehabilitation programs, is also necessary to maximize cardiovascular health and patient recovery [132]. However, the long-term use of these cardiovascular drugs can lead to adverse effects, such as muscle pain and liver toxicity (statins), fatigue and bronchospasm (beta-blockers), cough and hyperkalemia (ACE inhibitors), and bleeding risks (antiplatelets/anticoagulants). PCSK9 inhibitors may cause injection site reactions, while SGLT2 inhibitors increase the risk of urinary infections and ketoacidosis [133,134]. These concerns have driven interest in alternative therapies like nutraceuticals (omega-3 and polyphenols) and plant-derived compounds (berberine and resveratrol), which have garnered attention due to their bioactive compounds with potential cardioprotective properties [135,136]. These compounds exhibit diverse mechanisms of action, such as antioxidant, anti-inflammatory, antihypertensive, and lipid-lowering effects, making them attractive candidates for CVD management.

### 8.1. Flavonoids

Flavonoids, a diverse group of naturally occurring polyphenolic compounds, are widely distributed in the plant kingdom and possess significant antioxidant, anti-inflammatory, and cardioprotective properties [137]. They share a common 15-carbon skeleton comprising two aromatic rings linked by a heterocyclic ring, and are categorized into flavonols, flavanols, flavones, isoflavones, and anthocyanins [138]. Key flavonoids include quercetin, kaempferol, myricetin, rutin, hesperidin, and naringenin, each with distinct biological effects. Rich dietary sources include berries (anthocyanins), citrus fruits (hesperidin, naringenin), onions (flavonols), and tea, particularly black and green tea, which are abundant in total flavonoids [139,140].

Flavonoids modulate oxidative stress, inflammation, and endothelial dysfunction, key drivers of CVDs, making them promising therapeutic agents [141,142]. Oxidative stress, a major contributor to CVD pathogenesis, results from excess ROS, leading to endothelial dysfunction, vascular inflammation, and tissue damage [143]. Flavonoids mitigate oxidative stress by scavenging ROS, enhancing endogenous antioxidant defenses, and inhibiting ROS-generating enzymes such as xanthine oxidase and NADPH oxidase. Their phenolic rings neutralize ROS like superoxide and peroxynitrite, preventing cellular injury. Additionally, flavonoids activate nuclear factor erythroid 2-related factor 2 (Nrf2), which upregulates antioxidant enzymes such as superoxide dismutase (SOD), catalase, and glutathione peroxidase (GPx) [144]. They also prevent low-density lipoprotein (LDL) oxidation, a key factor in atherosclerosis [145].

Beyond antioxidation, flavonoids possess strong anti-inflammatory effects, inhibiting vascular inflammation by suppressing pro-inflammatory cytokines (IL-1β, TNF-α, IL-6, and IL-8) and enzymes such as cyclooxygenase-2 (COX-2) and lipoxygenase [146]. They improve endothelial function by enhancing NO bioavailability, promoting vasodilation, and reducing blood pressure [147]. Their anti-thrombotic effects prevent clot formation by inhibiting platelet aggregation and coagulation factor activation. Anti-atherogenic properties limit arterial plaque formation, reducing the risk of coronary heart disease [148]. By modulating the NF-κB, PI3K-AKT, and Nrf2/HO-1 pathways, flavonoids suppress inflammation, oxidative stress, and macrophage-mediated endothelial dysfunction, thereby preventing atherosclerosis and hypertension [149].

In myocardial infarction and ischemia–reperfusion injury, flavonoids offer cardioprotection via antioxidative, anti-inflammatory, and anti-apoptotic mechanisms [150]. Preclinical studies highlight the efficacy of morin and isoliquiritigenin in reducing myocardial damage and improving cardiac function [151]. Their antioxidant actions involve free radical scavenging and upregulation of SOD and catalase. Isoliquiritigenin activates Nrf2/HO-1 signaling, suppressing oxidative stress and ferroptosis in cardiomyocytes [152]. Flavonoids also downregulate the NF-κB and SAPK pathways (p38, JNK), reducing inflammation [153]. Their anti-apoptotic effects involve the regulation of survival pathways such as the RISK pathway (ERK, eNOS), with morin significantly inhibiting apoptosis in ischemia–reperfusion models [154]. Additionally, they modulate MAPK and NF-κB signaling, regulate calcium homeostasis, and protect mitochondria and autophagic pathways, enhancing cardioprotection [155]. However, clinical translation remains challenging, necessitating further research on their mechanisms and therapeutic interactions [155].

Flavonoids also influence mitochondrial function by activating mitochondrial potassium (mitoK) channels, which reduce calcium overload, stabilize membrane potential, and prevent ischemia–reperfusion-induced apoptosis [155,156]. Flavonoids like naringenin, quercetin, and epigallocatechin gallate enhance mitoK channel activity, reducing oxidative damage and supporting cardiovascular health [155]. Additionally, they improve vasodilation by enhancing endothelial nitric oxide synthase (eNOS) activity, reducing vascular stiffness, and lowering blood pressure [157]. Their inhibition of the angiotensin-converting enzyme (ACE) reduces vasoconstriction, further benefiting cardiovascular function [158]. Flavonoids prevent cardiomyocyte apoptosis by modulating apoptotic proteins, inhibiting JNK and p38 MAPK signaling, and promoting survival pathways like PI3K/Akt [159,160]. Their antioxidant actions prevent mitochondrial dysfunction by reducing ROS generation, maintaining membrane potential, and preventing mitochondrial permeability transition pore (MPTP) opening, which is particularly beneficial in diabetic cardiomyopathy and myocardial ischemia–reperfusion injury [161]. These broad molecular targets make flavonoids promising candidates for preventing oxidative stress-induced myocardial damage and CVD progression [162].

### 8.2. Omega-3 Fatty Acids

Omega-3 fatty acids, particularly eicosapentaenoic acid (EPA) and docosahexaenoic acid (DHA), play a crucial role in cardiovascular health [163]. A systematic review found that marine omega-3 fatty acids significantly reduce the risk of cardiovascular events by 10%, cardiac death by 9%, and coronary events by 18%, particularly in high-risk individuals [164]. These benefits stem from their ability to improve lipid profiles, reduce blood pressure, enhance vascular function, and exert anti-inflammatory effects [165]. Furthermore, omega-3 supplementation has been shown to lower the incidence of sudden cardiac death and all-cause mortality in post-myocardial infarction patients.

Current dietary recommendations advocate for at least two servings of fatty fish per week for the general population to maintain cardiovascular health. Individuals diagnosed with coronary heart disease are advised to consume approximately 1 g/day of EPA and DHA, while those with hypertriglyceridemia benefit from higher doses of 3–5 g/day under medical supervision to achieve significant triglyceride-lowering effects. Epidemiological studies consistently demonstrate that populations with high fish consumption exhibit lower rates of CVD morbidity and mortality [166]. Omega-3 fatty acids exert cardioprotective effects through multiple mechanisms. They regulate lipid metabolism by lowering triglyceride levels, inhibiting diacylglycerol O-acyltransferase (DGAT), and activating peroxisome proliferator-activated receptors (PPAR-α), leading to decreased triglyceride accumulation [167]. These effects are particularly beneficial for individuals with hypertriglyceridemia, a known risk factor for atherosclerosis. Beyond lipid metabolism, omega-3 fatty acids modulate inflammatory pathways that contribute to atherosclerosis. EPA and DHA inhibit the conversion of arachidonic acid into pro-inflammatory eicosanoids by downregulating cyclooxygenase-2 (COX-2) and lipoxygenase (LOX) enzyme activity [168]. Instead, they promote the production of specialized pro-resolving lipid mediators such as resolvins, protectins, and maresins, which reduce endothelial activation and suppress pro-inflammatory cytokines like interleukin-6 (IL-6) and tumor necrosis factor-alpha (TNF-α). This mitigates chronic vascular inflammation, helping to prevent atherosclerotic plaque formation and rupture [169,170].

Additionally, omega-3 fatty acids exhibit antithrombotic properties by reducing platelet aggregation and altering membrane phospholipid composition. They lower thromboxane A2 (TXA2) synthesis while increasing prostacyclin (PGI2) production, thereby reducing the likelihood of thrombosis [170,171]. Omega-3s also improve endothelial function by enhancing nitric oxide (NO) bioavailability, which promotes vasodilation, reduces arterial stiffness, and lowers blood pressure [172]. Moreover, omega-3 fatty acids influence cardiac electrophysiology, stabilizing sodium and calcium ion channels to reduce arrhythmias, a major cause of sudden cardiac death [173]. Clinical trials indicate that omega-3 supplementation significantly lowers the incidence of atrial fibrillation and ventricular arrhythmias, particularly in post-myocardial infarction patients. Despite strong observational and mechanistic evidence, some clinical trials have reported mixed results regarding omega-3 supplementation in CVD prevention [174], potentially due to variations in dosage, baseline omega-3 levels, genetic factors, and concurrent lipid-lowering therapies. Further research is needed to refine optimal dosing strategies and identify patient subgroups who may derive the greatest benefit. Nonetheless, the overall evidence supports the inclusion of omega-3 fatty acids in dietary and pharmacological strategies aimed at reducing CVD risk.

### 8.3. Resveratrol

Resveratrol, another polyphenolic stilbenoid found in grapes and peanuts, has shown significant cardioprotective action through various mechanisms, including antioxidant, anti-inflammatory, vasoprotective, and anti-atherosclerotic effects [175]. It reduces oxidative stress by inhibiting LDL oxidation and activating endogenous antioxidant enzymes, thereby preventing endothelial dysfunction and vascular damage [176]. Moreover, it enhances NO production, endothelial function, platelet aggregation inhibition, and reduced vascular inflammation that account for its vasoprotective effect [177]. Resveratrol is also very active in expressing anti-inflammatory activities by downregulating central inflammatory markers and enhancing the signaling cascade of SPMs, such as resolvins, that contribute to countering chronic inflammation in cardiovascular diseases [178]. It controls vascular smooth muscle cell (VSMC) function by suppressing lipid uptake and migration, thereby limiting atherosclerotic growth [179]. Resveratrol is also ischemia–reperfusion injury and cardiac hypertrophy protective, and its cardioprotective action is due to the activation of central molecular targets, including sirtuins (SIRT1), AMPK, and estrogen receptor α, which control oxidative stress, vascular function, and inflammatory responses [180]. Preclinical studies point to the potential of resveratrol in the prevention and treatment of hypertension, atherosclerosis, and heart failure, with synthetic analogs under investigation to enhance its bioavailability and clinical efficacy [181]. However, despite promising accounts, clinical evidence remains elusive due to concerns of bioavailability and study design but, at the same time, emphasizes the need for further studies in the formulation of optimal resveratrol-based interventions. As a result of the etiologically complex nature of cardiovascular disease, an integrated approach incorporating resveratrol with other therapies might be the most effective way of reducing cardiovascular risk [182].

### 8.4. Curcumin

The active compound of turmeric has predominantly cardiovascular protective effects through various mechanisms. It normalizes the levels of lipids by reducing total LDL cholesterol and triglycerides but increasing HDL cholesterol [183]. Through its potent antioxidant and anti-inflammatory activities, it inactivates ROS and regulates inflammation pathways, reducing oxidative stress and vascular inflammation, which are at the epicenter of the etiopathogenesis of CVD [184]. Curcumin possesses anti-thrombotic and anti-atherosclerotic effects by inhibiting platelet aggregation, preventing endothelial dysfunction, and slowing the development of atherosclerosis [185]. It is protective against drug-induced cardiotoxicity, such as adriamycin-induced damage, and inhibits ischemia–reperfusion (I/R) injury by stimulating SIRT1, which decreases myocardial damage and oxidative stress [186]. Additionally, curcumin exerts cardioprotective actions in diabetic cardiomyopathy (DCM) by activating SIRT1/FoxO1 and PI3K/Akt and the Nrf2/ARE pathway, inhibiting oxidative damage and improving cardiac function. In myocardial ischemia–reperfusion (MI/R) injury, it stimulates the JAK2/STAT3 pathway, enhancing cardiac repair mechanisms [187]. Additionally, curcumin delays cardiac aging by inhibiting the p53/p21 pathway, which prevents cellular senescence and oxidative damage [188]. Curcumin suppresses cardiac hypertrophy and heart failure by downregulating the p300-HAT enzyme that regulates cardiac remodeling, thereby attenuating hypertrophy and fibrosis [189]. Curcumin improves endothelial function and vascular health via increased NO bioavailability, improved arterial compliance, and reduced arterial stiffness, rendering it beneficial for hypertension and vascular disease [190]. Curcumin also controls metabolic syndrome by increasing insulin sensitivity, improving glucose metabolism, and facilitating weight regulation—major participants in averting the cardiovascular complications associated with diabetes [191]. Despite these favorable actions, curcumin’s therapeutic application is limited by its poor bioavailability. More recent formulations such as nano-curcumin and curcumin derivatives are under development to enhance its cardiovascular action.

### 8.5. Coenzyme Q10 (CoQ10)

This is a promising cardiovascular disease prevention and therapeutic drug due to its antioxidant, anti-inflammatory, and mitochondrial-supportive actions in enhancing cellular energy production and inhibiting oxidative stress [192]. As a potent antioxidant, CoQ10 can neutralize ROS that induce oxidative damage to lipids, proteins, and DNA, which is significant in the pathologies of cardiovascular diseases such as atherosclerosis and endothelial dysfunction [193]. In addition, CoQ10 improves mitochondrial function by increasing ATP synthesis, particularly in energy-demanding tissues such as the heart, while maintaining mitochondrial membrane stability and blocking apoptosis resulting from mitochondrial damage [194]. Its protective effects against vessels are also highlighted through its ability to improve endothelial function with increased nitric oxide (NO) bioavailability and reduced superoxide anion production, leading to increased vasodilation and lowered blood pressure [195]. Clinical evidence has shown the effects of CoQ10 supplementation on various cardiovascular illnesses, particularly heart failure, wherein trials like the Q-SYMBIO trial have revealed reductions in major adverse cardiovascular events and mortality [196]. For myocardial infarction, CoQ10 may enhance the survival of cardiomyocytes during ischemia and decrease remodeling following infarction, and antihypertensive effects have been proven using meta-analyses with a reduction in systolic and diastolic blood pressure [197]. The protective effect of CoQ10 against atherosclerosis is because it can inhibit the oxidation of LDL, reducing arterial plaque accumulation and improving vascular compliance [198]. Moreover, in diabetes, CoQ10 has been associated with improvements in lipid profiles, including reductions in LDL and total cholesterol levels, as well as potential impacts on glucose control [199]. While randomized controlled trials (RCTs) have been promising, particularly in improving systolic function and endothelial function, more large-scale clinical studies are needed to confirm the optimum dosage, duration, and long-term impact of CoQ10 supplementation in CVDs. Despite its superb safety record, CoQ10 can interact with anticoagulants like warfarin and may be of benefit in statin-treated patients as statins lower endogenous CoQ10 levels to cause myopathy [200,201]. Also, because variation in absorption, metabolism, and action of CoQ10 occurs between individuals, the personalized management of supplementation has to be used, and there is some evidence that the reduced form, ubiquinol, will yield superior bioavailability [202]. Overall, CoQ10 is a viable adjunctive therapy for cardiovascular disease, considering its ability to enhance mitochondrial bioenergetics, reduce oxidative stress, improve endothelial function, and support general cardiac health, and therefore additional clinical investigation of its applications is warranted.

### 8.6. Marine-Derived Compounds

These have gained huge attention regarding CVD prevention and management owing to their rich bioactive properties like antioxidant, anti-inflammatory, lipid-lowering, and antihypertensive activities. Marine microalgae, rich in polysaccharides, peptides, and carotenoids, have been reported to possess strong antioxidant and anti-inflammatory activity, which may protect against CVD by inhibiting oxidative stress and endothelial dysfunction [203]. Among the carotenoids derived from marine origins, astaxanthin and fucoxanthin stand out for their potential to scavenge free radicals, increase lipid metabolism, and increase the level of HDL cholesterol that exhibits major functions towards averting cardiovascular hazards [204]. Bioactive metabolites of marine origin include xyloketal B, asperlin, saringosterol, and omega-3 acid ethyl esters with cardioprotection, evidenced as acting through the major mechanisms for dealing with hypertension, ischemic heart disease, atherosclerosis, and myocardial infarction [205]. Phlorotannins, new seaweed polyphenols, possess promise regarding the modulation of CVD and metabolic disorder risk factors such as hyperglycemia, hyperlipidemia, inflammation, and oxidative stress, and they are potentially good candidates for the development of functional foods [206]. Seaweeds, collectively, are rich sources of bioactive compounds such as soluble dietary fiber, peptides, lipids, and minerals that have been shown to regulate lipid profiles, lower blood pressure, and improve vascular function, hence preventing CVD [207]. Fresh evidence also suggests the interactions among seafood-derived compounds, the gut microbiota, and cardiovascular health, highlight the influence of fish-derived proteins, peptides, and algal constituents on gut microbiome diversity, intestinal wall integrity, and systemic inflammation, which are crucial to CVD pathogenesis [208]. Despite strong preclinical and epidemiological evidence for the cardiovascular protective action of marine-derived products, variability among clinical trial results suggests that bioavailability, individual metabolic heterogeneity, dosage, and dietary interaction must be kept in mind to ensure the highest therapeutic benefit [209]. More large, well-designed clinical trials are essential to validate such findings and establish standardized protocols for the use of marine-derived constituents in CVD prevention and cure. Table 6 explains the various bioactive compounds with cardioprotective impacts.

## 9. Natural Products as Microbial Modulators for Cardioprotection

Natural products are potent microbial modulators with the potential to affect cardiovascular health by remodeling the gut microbiota environment [210]. The gut microbiota plays a crucial role in modulating cardiovascular homeostasis as well as in disease development, such as that of atherosclerosis, hypertension, and heart failure. Natural compounds can modulate the composition and function of the gut microbiota, inhibit systemic inflammation, regulate lipid metabolism, and improve the gut barrier, ultimately exerting significant cardioprotective effects [211].

Various bioactive natural compounds possess cardioprotective action through modulating the gut microbiota and host metabolic functions. Berberine, an isoquinoline alkaloid isolated from the *Coptis chinensis* and *Berberis* plants, promotes the growth of health-promoting SCFA-producing bacteria (*Roseburia*, *Blautia*, and *Alistipes*), strengthens intestinal barrier function, suppresses LPS-induced inflammation through TLR4/NF-κB, and improves lipid profiles, all providing overall cardiovascular protection [212]. Berberine improves lipid profiles by reducing serum cholesterol, triglycerides, and LDL levels, further protecting against CVD [213], while increasing high-density lipoprotein cholesterol (HDL-C) in individuals with hyperlipidemia.

Polymethoxyflavones (PMFs) from aged citrus peel support the increase in beneficial bacteria such as *Akkermansia* and *Bifidobacterium*, inhibit the production of TMA by targeting TMA-producing bacteria and hepatic FMO3, and decrease the NF-κB/MAPK signaling pathways, thus preventing vascular inflammation and atherosclerosis (AS) formation [214]. Resveratrol, a polyphenol found in grapes, modulates the gut microbiota by promoting beneficial bacteria like *Bacteroides*, *Lactobacillus*, and *Bifidobacterium*, which support gut homeostasis and reduce inflammation [215], while lowering pathogenic bacteria like *Enterococcus faecalis*, improving the bile acid metabolism, lowering TMAO, and exhibiting protective action against oxidative stress and endothelial dysfunction. It enhances endothelial function by increasing NO bioavailability, improving vascular health, and lowering the risk of atherosclerosis and hypertension. Its antioxidant and anti-inflammatory properties further protect against cardiovascular diseases by reducing oxidative stress and chronic inflammation [216]. While promising for gut and cardiovascular health, further clinical studies are needed to fully understand its therapeutic potential.

Moreover, quercetin, a flavonoid, possesses prebiotic functions by modulating gut microbiota, enhancing gut barrier function, and generating bioactive metabolites. It activates health-promoting bacteria, strengthens tight junctions to suppress inflammation, and generates antioxidant and anti-inflammatory metabolites that have beneficial effects on overall health [217]. Quercetin has prebiotic-like effects, which help restore gut microbiota after antibiotic exposure [218]. Thirty-two metabolic parameters, including BA biosynthesis intermediates 3,7,12,26-tetrahydroxy-5-cholestane (C27H48O4), are linked to its effect [218]. Quercetin indirectly regulates bile acid profiles through microbiota reshaping, contributing significantly to its anti-obesity and metabolic benefits in abdominal obesity-related metabolic syndrome (MetS) [219]. Quercetin also causes intestinal antioxidant enzyme upregulation and nutrient uptake transporters, reverses GM imbalance, and blocks inflammasome activation and TLR-4 signaling pathway stimulation [220]. Quercetin combined with resveratrol corrects HFD-induced dysbiosis by decreasing the density of pathogenic bacteria and the F/B ratio [221].

Ferulic acid (FA), a phenolic acid, exhibits anti-inflammatory, antioxidant, and cardioprotective effects. FA promotes lipid metabolism, inhibits the risk of thrombosis, and regulates diabetes [222]. FA decreases cholesterol, triglyceride, and LDL levels, and increases the F/B ratio in gut microbiota composition. FA regulates fat metabolism by promoting PPAR-α expression and inhibiting PPAR-β/γ expression [223]. In mice, FA reshapes the gut microbiota and fecal metabolites, diminishes pro-inflammatory cytokines (IL-1β, IL-2, IL-6, and TNF-α), decreases *Prevotellaceae*, and enhances beneficial *Lachnospiraceae* family abundance [224]. Other phytochemicals, such as apigenin and silibinin, also help by triggering SCFA production, regulating lipid metabolism and blocking oxidative stress and vascular inflammation, and thus promoting cardiovascular resilience [225].

Curcumin, the bioactive compound in turmeric, exerts anti-inflammatory and antioxidant effects by modulating the gut microbiota and promoting beneficial bacteria while suppressing harmful ones, thereby reducing systemic inflammation [226]. It strengthens the intestinal barrier, preventing the leakage of harmful substances like LPS into the bloodstream, which can trigger inflammation. Curcumin also inhibits pro-inflammatory pathways such as NF-κB and regulates lipid metabolism, lowering the risk of atherosclerosis and CVD [227]. These combined actions make curcumin a promising natural compound for gut and cardiovascular health.

Pomegranate juice is rich in polyphenols and exhibits anti-inflammatory properties [228]. Pomegranate juice significantly reduces atherosclerotic lesion size and intima-media thickness, decreases LDL uptake by macrophages, increases HDL levels by 27%, and lowers CVD risk by 12–18% [229]. Anthocyanins are flavonoid polyphenols that possess antioxidant activity and are associated with reduced CVD risk [230,231]. Anthocyanins favorably regulate the gut microbiota by increasing the counts of *Bifidobacterium*, *Lactobacillus*, *Roseburia*, *Akkermansia*, and *Parabacteroides* [232]. Anthocyanins enhance vascular function through enhanced production of NO, decreased oxidative stress, and inhibited aortic inflammation, leading to decreased leukocyte infiltration, reduced plaque formation, and decreased levels of inflammatory cytokines in the blood [233]. They also affect the NF-κB inflammatory pathway, liver lipid metabolism, and redox homeostasis [234]. In addition, anthocyanins also regulate gene expression linked to atherosclerosis in macrophages, endothelial cells, and the aorta, and evidence suggests that they regulate over 1200 genes [235]. Metabolites like protocatechuic acid also inhibit atherosclerosis by activating the miRNA-10b-ABCA1/ABCG1 cholesterol efflux pathway [236]. Nevertheless, although there is potential in the emphasis on natural products, it should be mentioned that not every microbial metabolite is beneficial; certain compounds, like LPS, are pro-inflammatory and are involved in cardiovascular disease. How these natural products are manifested in real-world environments where there is gut microbiota variability remains to be fully understood. Table 7 summarizes the role of natural products in modulating cardiovascular microbial activity.

## 10. Synergistic Effects of Phytochemicals in CVD

**Figure 2:**
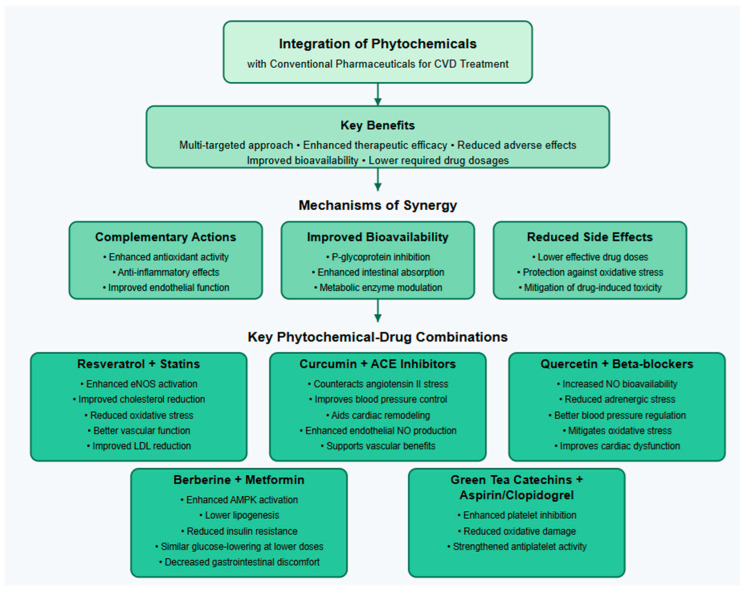
The central concept of phytochemical–pharmaceutical integration for CVD treatment.

The integration of phytochemicals with conventional pharmaceuticals offers a multi-targeted approach to managing CVDs and enhancing therapeutic efficacy while potentially mitigating adverse effects [237]. Phytochemicals interact with various molecular pathways, complementing the mechanisms of cardiovascular drugs [238,239]. Flavonoids, phenolic acids, and polyphenols, commonly present in fruits, vegetables, tea, and medicinal plants, have remarkable antioxidant and anti-inflammatory activity that can enhance the therapeutic efficacy of traditional drugs like statins, beta-blockers, and angiotensin-converting enzyme (ACE) inhibitors [240]. For instance, polyphenols like resveratrol and quercetin exhibit anti-inflammatory and antioxidant properties that enhance statin-induced cholesterol reduction [241,242], while curcumin and berberine improve endothelial nitric oxide production, supporting the vascular benefits of ACE inhibitors [243,244]. Many conventional drugs suffer from limited bioavailability, which can be improved through phytochemical co-administration. Curcumin enhances the intestinal absorption of statins by inhibiting P-glycoprotein efflux pumps, and piperine from black pepper increases metformin bioavailability, enhancing its glucose-lowering effects [245]. Phytochemicals may also reduce the required dosage of conventional drugs, minimizing side effects [243]. Berberine combined with metformin achieves similar glucose-lowering effects at lower metformin doses, reducing gastrointestinal discomfort [246], while quercetin co-administration with beta-blockers helps mitigate oxidative stress-induced cardiac dysfunction, improving patient compliance [247]. Clinical studies highlight the benefits of phytochemical–drug combinations in enhancing endothelial function, lipid metabolism, and glucose control. For example, resveratrol and statins improve endothelial nitric oxide bioavailability and reduce vascular inflammation [248], berberine combined with statins offers superior LDL cholesterol-lowering effects, and green tea catechins enhance the antiplatelet activity of aspirin while reducing gastric irritation [249]. Specific phytochemical-drug combinations with demonstrated synergistic effects include resveratrol with statins, which enhances endothelial nitric oxide synthase (eNOS) activation and reduces oxidative stress, improving LDL reduction and vascular function [250]; curcumin with ACE inhibitors, which counteracts angiotensin II-mediated oxidative stress, aiding blood pressure control and cardiac remodeling [244]; quercetin with beta-blockers, which increases nitric oxide bioavailability and reduces adrenergic stress [251], leading to better blood pressure regulation; berberine with metformin, which activates AMPK to mimic metformin’s effects while lowering lipogenesis and insulin resistance [252]; and green tea catechins with aspirin or clopidogrel, which enhance platelet inhibition and reduce oxidative damage, strengthening antiplatelet activity and lowering thrombotic risk [253]. Moreover, when used alongside statins, garlic supplementation has also been observed to increase the improvements in lipid profiles without increasing the risk of hepatotoxicity [254]. With continued scientific investigation and clinical approval, the combination of phytochemicals with conventional pharmacological treatments has the potential to revolutionize cardiovascular medicine, offering a more effective, safer, and holistic treatment paradigm for diseases of the heart. Figure 2 shows the integration of phytochemicals with conventional pharmaceuticals for CVD management.

## 11. Challenges and Future Directions

Although phytochemical–drug combinations show promising evidence in terms of treating CVDs, several initial problems discourage their implementation on a regular basis, including the inadequacies of large studies, regulatory problems, variation in the composition of phytochemicals, and herb–drug interactions [255]. Well-designed large-scale randomized clinical trials are needed to prove efficacy and safety before clinical application since most of them are observational or pilot-based with lower methodological rigor and short follow-up intervals [256], making it impossible to assess long-term cardiovascular effects. In addition, the heterogeneity of the patient population, genetic diversity, comorbid conditions, and food intake, also limit clinical evidence, necessitating larger and more heterogeneous study populations to be examined [257].

Mechanistic research that utilizes omics-based methods (metabolomics, proteomics, and transcriptomics) can supply clues on how phytochemicals interact with traditional drugs at the molecular level and further augment precision medicine practices [258]. Furthermore, regulatory and standardization issues as phytochemicals typically fail to meet requirements for quality control and dosage standardization demanded for drugs [259]. Plant species variation, cultivation practices, extraction methods, and storage methods all play a huge role in contributing to variations in the concentrations of bioactive compounds, making it difficult to achieve consistent therapeutic effects [260]. The regulatory classification issues are also part of the scenario, with most phytochemicals being classified as dietary supplements rather than pharmaceuticals and therefore restricted from use in evidence-based clinical care [261]. Pharmaceutical-quality material with defined bioactive content and stability must be formulated to enable regulatory approval and clinical use [262].

Another important issue is drug–herb interaction, since certain phytochemicals can influence drug metabolism through the inhibition of cytochrome P450 (CYP) enzymes and drug transporters such as P-glycoprotein (P-gp) [263]. For instance, grapefruit polyphenols inhibit CYP3A4, thereby increasing plasma concentrations and toxicity of statins and calcium channel blockers [264], whereas St. John’s Wort stimulates CYP3A4, reducing the efficacy of warfarin and beta-blockers [265]. Furthermore, curcumin and quercetin inhibit P-gp, affecting drug absorption; whereas, phytochemicals such as garlic, ginseng, and ginger exhibit anticoagulant activity that can enhance bleeding risks when co-administered with warfarin or aspirin [266]. Therefore, standardized protocols are required to guide clinicians on the safe co-administration of phytochemicals with cardiovascular drugs.

Pharmacogenomic factors add additional complexity to the clinical application of phytochemical–drug combinations because CYP450 enzyme polymorphisms (e.g., CYP2C19, CYP2D6, and CYP3A4) can influence drug metabolism and impact the efficacy of statins, beta-blockers, and antiplatelet drugs [267]. The genetic screening of response markers to phytochemicals may be able to forecast individual responses, and new evidence highlights the gut microbiota as the central player in phytochemical metabolism and the regulation of bioavailability and therapeutic effects [268]. Future research will have to center on microbiome-based precision medicine strategies to optimize phytochemical–drug interactions within clearly defined patient subgroups. Additionally, the development of personalized dosing regimens via genetic, metabolic, and gut microbiome profiling can potentially enhance efficacy and safety even further, leading towards the successful application of phytochemicals to mainstream cardiovascular medicine.

In summary, in the face of gigantic hurdles, progress in precision medicine, regulation standardization, and microbiome sciences offer hopeful fields to overcome today’s limitations. Future endeavors would have to include the incorporation of phytochemicals into evidence-based cardiovascular prevention and treatment in large, well-designed clinical trials, mechanistic studies, and the creation of patient-specified therapeutic regimens.

## 12. Conclusions

**Figure 3:**
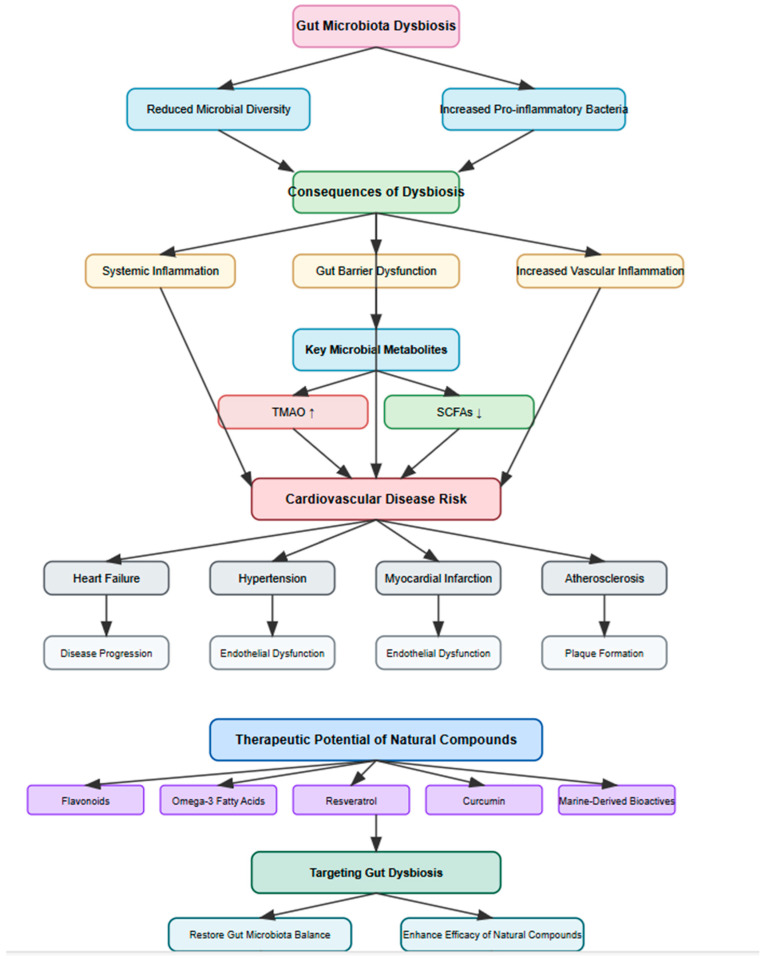
The complex relationship between gut microbiota dysbiosis and CVDs.

Growing evidence highlights the intricate relationship between gut microbiota dysbiosis and CVDs, including HF, hypertension, MI, and atherosclerosis. Dysbiosis contributes to systemic inflammation, vascular dysfunction, and disease progression through microbial-derived metabolites such as TMAO and SCFAs. The potential of natural compounds, including flavonoids, omega-3 fatty acids, resveratrol, curcumin, and marine-derived bioactives to restore microbial balance and confer cardioprotective effects presents a promising therapeutic avenue. Targeting gut microbiota offers novel strategies for CVD prevention and management, yet further research is needed to elucidate mechanistic pathways and develop microbiome-based interventions. Figure 3 shows the understanding the interplay between the gut microbiota and cardiovascular health may pave the way for personalized therapeutic approaches, ultimately improving patient outcomes.

## Figures and Tables

**Table 1:** The pathways of gut-derived metabolites orchestrate complex pathological changes during HF (↑: upregulation,↓: downregulation).

Metabolite	Activated Pathway	Key Effects	Impact on Heart Failure
SCFAs (acetate, propionate, butyrate)	GPR41, GPR43, GPR109A activation; HDAC inhibition	↑ IL-10 (anti-inflammatory cytokine), ↓ systemic inflammation, improved gut barrier integrity	Protective: maintains epithelial integrity, reduces microbial translocation, limits inflammation
TMAO	NF-κB activation; ROS/TXNIP/NLRP3 inflammasome; TGF-β1/Smad3 signaling	↑ Pro-inflammatory cytokines, ↑ oxidative stress, endothelial dysfunction, fibrosis	Detrimental: promotes atherosclerosis, cardiac dysfunction, and maladaptive remodeling
Bile Acids (BAs)	FXR activation; AIM2 inflammasome; Type I interferon pathways	Modulation of lipid metabolism, immune responses, mitochondrial dysfunction, cardiac hypertrophy	Detrimental: worsens inflammation, fibrosis, and metabolic derangement
BCAAs	mTOR pathway activation	↑ Oxidative stress, ↑ inflammation	Detrimental: exacerbates cardiac stress and injury
Tryptophan Derivatives (e.g., indole derivatives)	NLRP3 inflammasome modulation; PERK pathway activation	Immune regulation, cellular stress responses	Mixed: potential protective or harmful effects depending on balance
LPS	TLR4 signaling activation	↑ Cytokine release, oxidative stress, fibrosis, arrhythmias	Detrimental: drives systemic inflammation and cardiac remodeling

**Table 2:** The pathways of gut-derived metabolites orchestrate complex pathological changes during hypertension.

Metabolite	Activated Pathway	Key Effects	Impact on Hypertension
Short-Chain Fatty Acids (SCFAs)	GPR43 activation, inhibition of RAS, modulation of ORs (Olfr78, OR10J5)	Vasodilation, reduced inflammation, regulation of sympathetic activity, enhanced NO production	Lowers blood pressure; protective
Trimethylamine-N-oxide (TMAO)	NF-κB activation, vascular inflammation pathways	Increases vascular inflammation, endothelial dysfunction, platelet hyperresponsiveness	Elevates blood pressure; pro-hypertensive
Indole Derivatives (from Tryptophan)	Aryl hydrocarbon receptor (AhR) activation	Modulates immune responses, maintains vascular integrity	Generally protective; supports vascular health
Kynurenine	Immune and vascular modulation	Affects vascular tone and renal function	Variable, depending on pathway balance
Serotonin (5-HT)	Vascular and renal regulatory pathways	Modulates vascular contraction and renal function	Complex effects; can contribute to hypertension if dysregulated
Polyamines (Putrescine, Spermidine)	NO synthesis modulation	Regulates vascular tone through NO activation/inhibition	Can lower or raise blood pressure depending on balance
Lipopolysaccharides (LPS)	TLR4-mediated inflammatory signaling	Induces systemic inflammation, endothelial dysfunction	Promotes hypertension; detrimental

**Table 3:** The pathways of gut-derived metabolites orchestrate complex pathological changes during MI.

Metabolite	Activated Pathway	Key Effects	Impact on MI
Trimethylamine N-oxide (TMAO)	NF-κB activation; calcium signaling disruption; ROS generation	Promotes inflammation, oxidative stress, endothelial dysfunction, arrhythmia, atherosclerotic plaque destabilization	Increases MI risk, worsens myocardial injury and outcomes
Short-Chain Fatty Acids (SCFAs) (e.g., acetate, butyrate, propionate)	GPR41/43 activation; HDAC inhibition; eNOS activation	Suppress inflammation, improve gut barrier function, enhance vasodilation	Protective role; their reduction may impair MI recovery
Phenylacetylglutamine (PAGln)	Adrenergic receptor activation on platelets	Enhances platelet aggregation and thrombosis	Elevates thrombotic risk, worsens MI severity
Lipopolysaccharide (LPS)	TLR4-NF-κB activation; ROS generation	Induces systemic inflammation, oxidative stress, endothelial dysfunction	Exacerbates myocardial injury and adverse remodeling
Long-Chain Fatty Acids (LCFAs)	Metabolic dysregulation (lipid metabolism)	Promote platelet aggregation and thrombus formation	Potential biomarker for early AMI; contributes to MI initiation
Aromatic amino acid metabolites	Oxidative stress pathways	Increase ROS generation, influence infarct size	Worsen post-MI outcomes through increased oxidative damage

**Table 4:** The pathways of gut-derived metabolites that orchestrate complex pathological changes during atherosclerosis.

Metabolite	Activated Pathway	Key Effects	Impact on Atherosclerosis
Trimethylamine N-oxide (TMAO)	TMAO pathway, MAPK, NF-κB signaling, inhibition of reverse cholesterol transport	- Impairs nitric oxide (NO) bioavailability, leading to endothelial dysfunction. - Enhances foam cell formation. - Increases oxidative stress and inflammation.	- Promotes atherosclerosis by causing endothelial dysfunction, plaque instability, and elevated cholesterol deposition.
Short-Chain Fatty Acids (SCFAs)	PPARγ activation, gut barrier integrity regulation	- Regulates inflammation. - Maintains gut integrity by tightening the gut lining. - Regulates lipid metabolism and reduces systemic inflammation.	- Protects against atherosclerosis by preventing LPS translocation and reducing vascular inflammation.
Lipopolysaccharides (LPS)	TLR4 signaling, NF-κB pathway	- Activates inflammatory response. - Increases endothelial activation and immune cell recruitment.	- Promotes endothelial dysfunction and accelerates plaque formation due to systemic inflammation.

**Table 5:** Overall types of CVD-associated gut dysbiosis.

Feature	Healthy Gut Microbiota	Atherosclerosis-Associated Dysbiosis	Hypertension-Associated Dysbiosis	Heart Failure-Associated Dysbiosis	Myocardial Infarction (MI)-Associated Dysbiosis
Microbial Diversity	High microbial richness and diversity	Reduced diversity, enrichment of pro-inflammatory species	Lower diversity, increased pathobionts	Significant microbial imbalance, loss of beneficial bacteria	Decreased diversity, dominance of pro-inflammatory bacteria
Bacillota/Bacteroidota Ratio	Balanced ratio, supporting homeostasis	Increased Bacillota/Bacteroidota ratio (linked to inflammation)	Increased Bacillota, decreased Bacteroidota	Elevated Bacillota levels, disrupting metabolic pathways	Increased Bacillota dominance, linked to inflammation
SCFA-Producing Bacteria	High levels of *Faecalibacterium*, *Roseburia*, *Akkermansia*	Decreased *Faecalibacterium*, *Roseburia*	Reduced *Akkermansia muciniphila*, impairing gut barrier	Loss of *Butyrivibrio* and *Faecalibacterium*	Decreased *Roseburia* and *Akkermansia*
SCFA Levels (Butyrate, Acetate, Propionate)	High, maintaining gut and vascular health	Decreased SCFA levels, leading to endothelial dysfunction	Lower SCFA levels, contributing to vascular stiffness	Markedly reduced SCFAs, worsening systemic inflammation	Reduced SCFAs, promoting pro-thrombotic environment
Inflammatory Bacteria	Low levels of *Escherichia*, *Enterobacter*	Increased *Escherichia coli*, *Enterobacter*, *Proteobacteria*	Overgrowth of *Desulfovibrio* (H2S producer, causing damage)	Elevated *Klebsiella*, *Enterococcus*, *Staphylococcus*	Increased *Enterobacter* and *Fusobacterium*
LPS-Producing Bacteria	Low levels, preventing endotoxemia	Increased *Klebsiella*, *Parabacteroides*	Enrichment of *Proteobacteria*, elevating systemic LPS	High *Enterobacteriaceae*, promoting systemic inflammation	Increased *Bacteroides* and *Proteobacteria*
TMAO-Producing Bacteria	Low levels, reducing cardiovascular risk	Enrichment of *Lachnoclostridium*, *Desulfovibrio* (TMAO producers)	Increased *Clostridia* and *Fusobacterium*, promoting TMAO	Elevated *Eggerthella lenta* and *Desulfovibrio*	Increased *Lachnospiraceae* and *Clostridium*
Bile Acid Metabolism	Normal bile acid balance, supporting lipid metabolism	Increased secondary bile acids (pro-inflammatory effects)	Altered bile acid conversion, affecting BP regulation	Elevated toxic bile acids, worsening cardiac function	Disrupted bile acid homeostasis, impairing heart recovery
Inflammatory Markers	Low levels of IL-6, TNF-α, CRP	Elevated IL-6, TNF-α, CRP, promoting plaque formation	Increased pro-inflammatory cytokines (IL-17, TNF-α)	High IL-6, TNF-α, gut permeability worsens	Increased IL-1β, IL-18, contributing to clot formation
Endothelial Function	Intact vascular endothelium, healthy BP regulation	Endothelial dysfunction, leading to atherosclerotic plaque	Reduced nitric oxide (NO), causing vascular constriction	Endothelial damage, exacerbating heart failure risk	Vascular inflammation, increasing thrombosis risk
Gut Barrier Integrity	Strong tight junctions, preventing microbial translocation	Impaired barrier, microbial translocation fuels inflammation	Weakened barrier, increasing BP-related damage	Severe gut leakiness, endotoxemia worsens heart function	Increased permeability, leading to systemic inflammation

**Table 6:** The key bioactive compounds with cardioprotective effects.

Compound	Sources	Cardioprotective Mechanisms	Key Molecular Targets	Clinical Implications	References
Flavonoids	Berries, citrus fruits, onions, tea	Antioxidant, anti-inflammatory, anti-atherosclerotic, anti-thrombotic, improves endothelial function, reduces blood pressure	Nrf2, NF-κB, PI3K-AKT, eNOS, COX-2, NADPH oxidase, MAPK	Potential role in preventing atherosclerosis, hypertension, and MI; needs further clinical translation	[137,138,139,140,141,142,143,144,145,146,147,148,149]
Omega-3 fatty acids	Fatty fish (EPA, DHA), flaxseeds, walnuts	Reduces triglycerides, lowers blood pressure, is anti-inflammatory, improves endothelial function, stabilizes cardiac electrophysiology	PPAR-α, COX-2, LOX, resolvins, prostacyclin, NO	Beneficial in reducing cardiovascular events, improving lipid profiles, and managing hypertriglyceridemia	[163,164,165,166,167,168,169,170,171,172,173,174]
Resveratrol	Grapes, peanuts, berries	Antioxidant, anti-inflammatory, anti-atherosclerotic, vasoprotective, inhibits LDL oxidation, enhances NO production	SIRT1, AMPK, estrogen receptor α, NF-κB, eNOS	Potential in managing hypertension, atherosclerosis, and heart failure; concerns regarding bioavailability	[175,176,177,178,179,180,181,182]
Curcumin	Turmeric, ginger	Reduces oxidative stress, is anti-inflammatory and anti-thrombotic, regulates lipids, inhibits platelet aggregation, prevents atherosclerosis	SIRT1, NF-κB, COX-2, NADPH oxidase	Cardioprotective in conditions like ischemia–reperfusion injury, diabetic cardiomyopathy, and drug-induced toxicity	[183,184,185,186]
Coenzyme Q10 (CoQ10)	Meat, fish, nuts, spinach, broccoli, whole grains	Antioxidant, mitochondrial function support, reduces oxidative stress, enhances energy production, improves endothelial function	Cytochrome c oxidase, Nrf2, ATP synthase, SIRT1	Potential benefits in heart failure, ischemic heart disease, hypertension, and statin-induced myopathy	[187,188,189,190,191,192,193]
Marine-derived compounds	Fish oils, seaweed, marine algae, krill oil	Anti-inflammatory, antioxidant, reduces cholesterol and triglycerides, protects against ischemic damage, improves endothelial health	PPAR-α, SIRT1, eNOS, COX-2, TLR4	Protective effects in cardiovascular diseases like atherosclerosis, heart failure, and inflammation	[194,195,196,197,198,199,200]

**Table 7:** Some natural products that modulate cardiovascular health through gut microbiota interactions.

Natural Product	Gut Microbiota Modulation	Cardiovascular Benefits	Mechanism of Action	References
Berberine	Promotes SCFA-producing bacteria (Roseburia, Blautia, Alistipes)	Reduces cholesterol, triglycerides, and LDL; increases HDL-C	Increases beneficial bacteria, suppresses LPS-induced inflammation via TLR4/NF-κB, improves lipid profiles and strengthens intestinal barrier function	[212,213]
Polymethoxyflavones (PMFs)	Increases Akkermansia and Bifidobacterium; inhibits TMA-producing bacteria	Prevents vascular inflammation and atherosclerosis; reduces TMAO	Inhibits TMA production, reduces NF-κB/MAPK signaling, improves gut health and prevents atherosclerosis	[214]
Resveratrol	Promotes Bacteroides, Lactobacillus, Bifidobacterium; reduces Enterococcus faecalis	Lowers risk of atherosclerosis and hypertension; improves endothelial function	Modulates gut microbiota to enhance NO bioavailability, reduces inflammation and oxidative stress, improves bile acid metabolism, and lowers TMAO	[215,216]
Quercetin	Activates health-promoting bacteria; restores gut microbiota after antibiotic exposure	Anti-obesity, metabolic benefits in metabolic syndrome; reduces inflammation	Strengthens tight junctions, generates antioxidant metabolites, regulates bile acid profiles, blocks inflammasome activation and TLR4 signaling pathway	[217,218,219,220]
Ferulic Acid (FA)	Increases Lachnospiraceae; reduces Prevotellaceae	Lowers cholesterol, triglycerides, LDL; promotes fat metabolism	Reshapes gut microbiota, activates PPAR-α, inhibits PPAR-β/γ, decreases pro-inflammatory cytokines, and regulates lipid metabolism	[222,223,224]
Curcumin	Promotes beneficial bacteria; suppresses harmful bacteria	Reduces inflammation and oxidative stress; lowers atherosclerosis risk	Inhibits NF-κB pathway, strengthens intestinal barrier, modulates lipid metabolism, reduces vascular inflammation, and prevents endotoxemia	[226,227]
Pomegranate Juice	Enhances Bifidobacterium, Lactobacillus, Roseburia, Akkermansia	Reduces atherosclerotic lesions; increases HDL-C and lowers CVD risk	Modulates gut microbiota, reduces atherosclerotic plaque size, enhances NO production, reduces oxidative stress and inflammatory cytokines	[228,229]
Anthocyanins	Increases Bifidobacterium, Lactobacillus, Roseburia, Akkermansia	Enhances vascular function; reduces plaque formation and aortic inflammation	Enhances NO production, decreases oxidative stress, modulates NF-κB pathway, regulates gene expression linked to atherosclerosis, improves redox homeostasis	[230,231,232,233,234,235]

## Data Availability

Data sharing is not applicable.
